# Editorial Notes

**Published:** 1932

**Authors:** 


### Editorial Notes

**Resignations of Professors Hey Groves and Rayner.**

At the end of this session the Chairs of Surgery and Obstetrics will be vacated by Professor Hey Groves and Professor Rayner respectively. Their retirement takes place under the University regulation which makes the limit of occupancy of the Chairs in clinical subjects coincide with resignation from the active staff of the constituent hospitals. Professors Groves and Rayner, having been elected to the Consulting Staff of the Bristol General Hospital, come under the operation of this regulation, and it is with great regret that the Medical School will part with two such valuable teachers, both of whom are Bristol alumni.

Professor Rayner received the whole of his medical education at the Bristol University College and the Bristol General Hospital. Having taken his F.R.C.S. England, he was appointed Assistant Physician Accoucheur at the Bristol General Hospital in 1897, being promoted to the full staff on Dr. Newnham's retirement in 1923. In 1926 he was elected to the University Chair of Obstetrics in succession to the late Professor Walter Swayne. To the practical value of his teaching many generations of students can testify, for he was an outstanding teacher at the Bristol General Hospital long before he was called to occupy the University Chair. He has had an even wider field of usefulness as a consulting obstetrician and gynæcologist. Bernard Shaw says in the preface to one of his plays: "The man who can, does: the man who cannot, teaches." He overlooked the possible existence of men like Rayner who, because they can do, are made to teach.

Professor Hey Groves commenced his studies in Bristol, and then went to Bart's. In 1905 he won the Gold Medal in the London M.S., and obtained the F.R.C.S. He was appointed to the Bristol General Hospital staff in 1903 and to the Chair of Surgery in 1922. His achievements in surgery are known all the world over, and he has been signally honoured by the Royal College of Surgeons by being elected to the office of Vice-President.

It is a matter for congratulation that both these Professors should have retired from the routine and exacting work of the University and Hospital in full health and still youthful vigour. They will have additional time to devote to the calls of private consulting practice, and their ripe experience and skill will in future be no less, but even more, at the disposal of the public and the medical profession. This is one of the compensations which an exceptionally early retiring age at the Bristol Hospitals and University carries with it.

**Branch Meeting of British Medical Association at Taunton.**

THE newly-constituted "Bath, Bristol and Somerset" Branch of the British Medical Association held its first meeting at Taunton on 29th June, in the Taunton and Somerset Hospital. Mr. W. G. Mumford, the President, was in the Chair, supported by twenty-four members. The President in opening the meeting expressed his hope that the new associations of this

Branch would prove prosperous and fortunate. Mr. J. R. Nicholson-Lailey, F.R.C.S., surgeon to the hospital, read a paper on "The Surgical Treatment of Peptic Ulcers," which it is hoped to publish in this *Journal* in the near future. The animated discussion evoked by the paper leaves no doubt that the extension of the Branch to include the whole county of Somerset will add greatly to its activity and interest. The meeting opened at 3.30 p.m. and closed shortly after 5 o'clock, tea being then served in the hospital. The success of this meeting will certainly increase the popularity and influence of the British Medical Association in the area which the Branch covers.

**British Pharmacopœia (1932).** THE following letter has been received from the Registrar of the General Medical Council :—

DEAR SIR,

The new issue of the *British Pharmacopœia* (1932) will be published on the 30th September, 1932. I am instructed, on behalf of the Council, to say that the Council wholly reserve their copyright in the new work ; and that nothing in the nature of substantial extracts from it must be published without the Council's express sanction. A memorandum relating to this matter in detail can be supplied on application. At the same time, no objection will be taken to such reasonable reference to the facts and figures therein set forth as may be properly made for purposes of criticism, review, and summary, or of study and research, but no information of any kind derived from the new issue may be published in book form before the 30th September, 1932.

Advance copies of the text of the *British Pharmacopœia* (1932) will be accessible on the 4th July, 1932, at this Office, and at 12 Queen Street, Edinburgh, and 35 Dawson Street,

Dublin, for the inspection of the public, from 10.0 a.m. to 1.0 p.m., and from 2.0 p.m. to 4.0 p.m. daily, excluding Saturdays, in order to give to manufacturers, pharmacists and others interested an opportunity of ascertaining the changes that are about to be made in official requirements, and of making the necessary arrangements to meet them.

Every person who takes advantage of this opportunity will be expected to observe the conditions above indicated, and will be required to sign an undertaking to that effect.

Inquiries by letter or telephone as to details of the contents of the new *Pharmacopœia*, or the changes about to be made in official requirements by its issue, cannot be answered. The advance copies themselves must be consulted.

I shall be obliged if you will insert this letter in your next issue.

Yours faithfully,

NORMAN C. KING,

*Registrar.*

*** The Medical Council Act, 1862, provides that :—

" The exclusive right of publishing, printing and selling the said *Pharmacopœia* shall vest in the said General Council."

The Copyright Act, 1911, may also be cited in this connexion :—

" 2. (1) Copyright in a work shall be deemed to be infringed by any person who, without the consent of the owner of the copyright, does anything the sole right to do which is by this Act conferred on the owner of the copyright : provided that the following acts shall not constitute an infringement of copyright :—

" (1) Any fair dealing with any work for the purposes of private study, research, criticism, review, or newspaper summary."

